# Supplementary material for: Oligo-FISH of Populus simonii Pachytene Chromosomes Improves Karyotyping and Genome Assembly
Source: Int J Mol Sci. 2023 Jun 9;24(12):9950. doi: 10.3390/ijms24129950 (PMC10297927; doi:10.3390/ijms24129950)
Supplement: Supplementary file 1 [file ijms-24-09950-s001.zip › ijms-2398244-supplementary.pdf]

Oligo-FISH of *Populus simonii* Pachytene Chromosomes Improves Karyotyping and Genome Assembly

Supplementary Materials

Supplementary Data S1. The sequence data of a centromere-specific repeat Ps34.

>Ps34

ACCGAGATCTACACAGGCGAAGACACTCTTTCCCTACACGACGCTCGCACAAACTTGGGG
AAGATGTAATCAACGCTGTTAGATCCTTCTTTCAGACTCGTAGAATGCTTAAAGAAACGAATGC
TACATCCATTTATTTTTTTTTTTTTGGTAAGTTGACGTATGTATTAAAAGAAGAAGATACAAACAT
TATAAGGGGCATACCCCAGATTTACAATGAGAACAGAAACATAAAACAACAGATGGCTACAA
GCTAGCCACCGACCATAAAACTGAAACCTCACTAGATTCGTTAGGGAAACCAATACAACCAAC
TAACTAACAAATTAAGAATCTATGGTATTCACCCTCCAAGCTTGTTGGATCCTTTTGTTTTCATTT
GTTGGTACGACATTCCTCATCAAATCAAGCTTATCACGAATGATATATTCGATTTGATTAAAGAC
CAAATTCGAAGTTCTAGACATTCCAGCAAAGATCCTTGCATTCCGTTCTTGCCATACATGATACA
CTGTAGCTGCGAAACTCAGTTTTCGAGAAAAATTGACGAAACTCTTGCCATGCCAAGAGACAGT
GGCCCATCGAATCCATTCATCCCAGCCTTTTGTCATTCTTGGAATGTCGCATCTGTCACAAACAT
CCCACCAGATCGCTTTAGTATAGGAGCATTCAAAGAACAAGTGGTTGTGATCCTCATTGTTGCG
GAGACAGAGTGAGCATCTATTGGGACCATGTATACCAAACCGATGAAGTTTATCTTGAGTTGTG
AGTTTCTGTTGGACAGCCATCCATAGAAGAAATGAATGTCTTGGAACAGCATTCTTGAACCACA
CAATGTCATGCCATTCAACCATCTGACGATGACCTCTTAGTTGTTCCCAAGCTACTTTGATCGAG
AATCTGTGATTTGGCGAATCCAACCAAACTATCTCATCCTTTTGCCCCATCTTAGGATTAGAATT
GGAAGGAATAGCTTCTATAATGGGGTGCCAGCCAATAGCTTGGGTGGTAGGAGTTTTCCATTCT
GAGTTATGAATTAGCACTTTCACCCTCGCGTTCTTGGCCATACCTGAATCATAGATAAATCTTTC
CCCGTAAGAATCCGCGAGTGGGCTGTGAGGATGCCAATTGTCAAACCATAGAGAGGTTGTCATT
CCATCTCCTATGATGTACTTCATCTTCGGCCATGCTAAGGATCTGAGCTTTAGAATCTTTCCCCA
AGCCCAAGAGCAATTCTGTGGCGTCTTGATTGTCCAGAAATTCCTACCTCGCAACAGATTGGAT
CTGATCCAAGTAGACCATATTGAGCCATCTGAGTCATTGCACAGGTTCCAAATGTGTTTCAACA
AAGCAATCTTGTTCCATTCTGTTATCCTTTTTATTCCTAGCCCCCCCTCCTTTTTTGGAAGACATAC
CTGATCCCAAGCCACTTTAGCCCCAGTAGTTCTCATATCTGAACCTGACCAAAGAAAGGATTTC
ATAATTTGCTCCACATTTTTAATTACTTGCCCAGGTAAGAGAAAGAGAGATGCCCAATAGACCT
GTATGGAAAATAAGACTGAATTAATCAGTTGTACCCGTCCTGCATACGAGAGTGTTCTACAAGT
CCAATGTCGAACTTTAGAGGTGATTCGATCCACGAGGCCCTTACAATAAACAGCCTTTAGTCTG
GACGAGAGAAGAGGAACTCCCAAATATTTCATAGGGAGCTCCCCCTCTCTAAACCCAAGAATA
TGAATAATTTGTTCCCTCTCAGCATTTAACACACCGCTCAAGAAGATGTCACTTTTGTTTGGATTT
GGATACAGACCTGATAGGTCTTGAAACTTTGTGAGCACAGTTCTGATCATACGAATTGAGTTTA
CATCCCCGTTGCTAAAAATCATCAAGTCGTCAGCAAAACAAAGATGAGAAATTTTGTCCTTCTT
GCATCTCCAGTGGAACTTGAATTCTTGGTTGGCACTCATCTTGCAGAATAGCCCCGAAAGGATTT
CCATACATAGGACAAACAAATATGGGGACAATGGATCCCCTTGTCTCAGCCCTCTTCCCCCTTG
AAAGTAACCTGCAAGCTCACCATTGACGTTGATAGAGAATTGACATGATGTAACACAAACCAT

GATCCAATCAATTACTGTTCTAGGGAATCCCATTTTTATTAACACAGCGTCAACGAAATCCCAC
CGCACCGAGTCGTAAGCCTTCATCAGATCAACTTTCATAGCACAACGAGCAGGTCCCGTAGATT
TATGATAGCCTTTCATTAGTTCCTGAGACAACAGAATGTTGTCACTGATTCTCCGTCCTGAGATA
AAAGCAGTCTGATATGGACCAACTAAGGATGGCAAAACAACTTTGATTCTCCCAGCTAGAATCT
TAGCGATGCATTTGTACACTGTATTGCAGCAAGATATAGGACGAAAATCTGTCAACCTTGTAGG
ATTAGCAACTTTAGGAATAAGGGAAATGGATGTAGCATTCATTTCTTTAAGCATTCTACGAGTCT
GAAAGAAGGATCTAACAGCGTTGATTACATCTTCCCCAACTATGTGCCACATTCTTTTGAAGAA
ACCTGCGTTAAAGCCATCTGGACCAGGGGCTTTGTTGTTCTTCAAACTAAACATAGCATGCTTAA
TCTCCTCCCTTGTTACATCTTGTGCGAGTACTTG
